# Partially Defective Store Operated Calcium Entry and Hem(ITAM) Signaling in Platelets of Serotonin Transporter Deficient Mice

**DOI:** 10.1371/journal.pone.0147664

**Published:** 2016-01-22

**Authors:** Karen Wolf, Attila Braun, Elizabeth J. Haining, Yu-Lun Tseng, Peter Kraft, Michael K. Schuhmann, Sanjeev K. Gotru, Wenchun Chen, Heike M. Hermanns, Guido Stoll, Klaus-Peter Lesch, Bernhard Nieswandt

**Affiliations:** 1 Institute of Experimental Biomedicine, University Hospital of Würzburg, Würzburg, Germany; 2 Rudolf Virchow Center, University of Würzburg, Würzburg, Germany; 3 Department of Psychiatry, Taipei Tzu Chi Hospital, Buddhist Tzu Chi Medical Foundation, Taipei, Taiwan; 4 Department of Neurology, University Hospital of Würzburg, Würzburg, Germany; 5 Department of Molecular Psychiatry, University Hospital of Würzburg, Würzburg, Germany; 6 Department of Medical Clinic and Policlinic II, Hepatology, University Hospital of Würzburg, Würzburg, Germany; Penn State Hershey College of Medicine, UNITED STATES

## Abstract

**Background:**

Serotonin (5-hydroxytryptamin, 5-HT) is an indolamine platelet agonist, biochemically derived from tryptophan. 5-HT is secreted from the enterochromaffin cells into the gastrointestinal tract and blood. Blood 5-HT has been proposed to regulate hemostasis by acting as a vasoconstrictor and by triggering platelet signaling through 5-HT receptor 2A (5HTR2A). Although platelets do not synthetize 5-HT, they take 5-HT up from the blood and store it in their dense granules which are secreted upon platelet activation.

**Objective:**

To identify the molecular composite of the 5-HT uptake system in platelets and elucidate the role of platelet released 5-HT in thrombosis and ischemic stroke. Methods: 5-HT transporter knockout mice (*5Htt*^*-/-*^) were analyzed in different *in vitro* and *in vivo* assays and in a model of ischemic stroke.

**Results:**

In *5Htt*^*-/-*^ platelets, 5-HT uptake from the blood was completely abolished and agonist-induced Ca^2+^ influx through store operated Ca^2+^ entry (SOCE), integrin activation, degranulation and aggregation responses to glycoprotein VI (GPVI) and C-type lectin-like receptor 2 (CLEC-2) were reduced. These observed *in vitro* defects in *5Htt*^*-/-*^ platelets could be normalized by the addition of exogenous 5-HT. Moreover, reduced 5-HT levels in the plasma, an increased bleeding time and the formation of unstable thrombi were observed *ex vivo* under flow and *in vivo* in the abdominal aorta and carotid artery of *5Htt*^*-/-*^ mice. Surprisingly, in the transient middle cerebral artery occlusion (tMCAO) model of ischemic stroke *5Htt*^*-/-*^ mice showed nearly normal infarct volume and the neurological outcome was comparable to control mice.

**Conclusion:**

Although secreted platelet 5-HT does not appear to play a crucial role in the development of reperfusion injury after stroke, it is essential to amplify the second phase of platelet activation through SOCE and plays an important role in thrombus stabilization.

## Introduction

Serotonin is an important neurotransmitter that has key functions within the brain and in the periphery. Apart from the brain, 5-HT is synthetized from tryptophan by tryptophan hydroxylase 1 (TPH1) by the enterochromaffin cells of the gut [1]. Decreased 5-HT uptake in the gut enhances intestinal inflammation [2] indicating that extracellular free 5-HT is an important inflammatory mediator and indeed, abolished 5-HT synthesis protects the gut from inflammation [3]. Mast cells, dendritic cells, NK cells and B cells express 5-HT receptors on the cell surface. Therefore secreted platelet 5-HT or increased 5-HT level in the blood can modulate their effector functions through 5-HT receptor signaling [4, 5]. Furthermore, secreted platelet 5-HT can act as a chemoattractant factor of mast cells, eosinophils and neutrophils at the site of acute inflammation [6]. In line with these results, inhibition of 5-HT uptake by selective serotonin re-uptake inhibitors (SSRI) results in suppression of pro-inflammatory cytokine expression [7]. Platelets are the biggest store of peripheral 5-HT and represent the major source of 5-HT for immune cells. Platelets cannot synthetize 5-HT [8], but they can take 5-HT up from the plasma through the serotonergic transport system [9], store it in dense granules [10], and release it into the blood during degranulation [11]. Extracellular or platelet released 5-HT can act on the platelet serotonin receptor, 5-HTR2A, and induces Gq mediated PLCβ activation which leads to the increase production of inositol triphosphate (IP_3_) and diacylglycerol (DAG). These second messengers can further modulate Ca^2+^ release from the store and enhances Ca^2+^ influx through the store-operated Ca^2+^ channel Orai1 and the DAG sensitive receptor-operated Ca^2+^ channel TRPC6 [12]. Although 5-HT alone can induce shape change of platelets, it potentiates aggregation only in combination with other platelet agonists [13]. The function of the major 5-HT transporter 5HTT can be enhanced or inhibited on the platelet surface. Its activity is important to maintain a store of 5-HT in platelet dense granules despite the concentration gradient of 5-HT between platelets and the external milieu [14, 15].

Glycoprotein VI (GPVI) is the major collagen receptor expressed on the surface of platelets with a signalosome consisting of the Fc receptor γ-chain, Src family kinase Syk and the linker for activation of T cells (LAT) which triggers Ca^2+^ store release through phospholipase C (PLC)γ2 activation. 5HTT is phosphorylated by Syk and thereby indirectly connected to the GPVI signalosome [16, 17]. Interestingly, decreased responses to the GPVI agonists and impaired secretion responses to collagen have been described in SSRI treated platelets [18]. The platelet store of 5-HT, the regulation of its uptake and levels in the periphery are often affected by SSRI used in the treatment of many cognitive disorders including depression [19]. SSRI treatment in patients with depression decreases 5-HT uptake by platelets [20] thereby having a protective effect against myocardial infarction [21], but it can also induce bleeding complications indicating that long-term blockage of 5-HT uptake system affects primary hemostasis [22, 23]. Although the blockage of 5-HT uptake in human platelets by SSRI treatment has been described [24], the direct function of the transporter 5HTT and platelet stored 5-HT in the context of hemostasis, thrombosis and stroke has not been studied. In the context of thrombosis, several *in vivo* studies have demonstrated the importance of peripheral 5-HT using *Tph1*^*-/-*^ mice or using *Wt* mice infused with 5-HT [25]. *In vivo* 5-HT infusion generates hyperreactive platelets with reduced bleeding times and shortened occlusion times of the carotid arteries in *Wt* mice [26]. 5-HT can both dilate and constrict the coronary vessels depending on the presence or absence of a normal endothelium, respectively [27]. Intra-coronary platelet deposition and 5-HT release can trigger a marked local vasoconstriction of large coronary arteries [28]. Interestingly, SSRI treatment has a significant impact on post-stroke recovery, but controversial results were published on infarct volume in animal models of ischemic stroke [29, 30]. In *5Htt*^*-/-*^ mice, elevated extracellular 5-HT levels were observed in the brain [31] which may trigger inflammatory cell migration under ischemic events, but the process of ischemia-induced thrombo-inflammation has not yet been investigated in these mice.

To study the uptake and release of 5-HT by platelets and its role in thrombosis and hemostasis, we have used a constitutive knockout of 5-HT transporter (*5Htt*^-/-^) mouse strain [32–34]. Our results identify 5HTT as the major route of 5-HT uptake in platelets and show that platelet stored 5-HT plays an important role in (hem)ITAM signaling and SOCE thereby influencing thrombosis and hemostasis.

## Materials and Methods

Thrombin (Roche Diagnostics), adenosine diphosphate (ADP, Sigma-Aldrich), high-molecular-weight heparin (Sigma-Aldrich), human fibrinogen (Sigma-Aldrich), U46619 (Alexis Biochemicals), collagen (Horm Kollagen, Nycomed), apyrase (Amersham/GE Healthcare), thapsigargin (TG, Invitrogen), Fura-2/AM (Invitrogen), and Pluronic F-127 (Molecular Probes), IP_1_ ELISA kit (Cisbio Bioassays) were purchased. Monoclonal antibodies conjugated to fluorescein isothiocyanate (FITC), phycoerythrin (PE), or DyLight-488 and the antibody against the activated form of integrin αIIbβ3 (JON/A-PE) were from Emfret Analytics (Eibelstadt, Germany). Collagen-related peptide (CRP) was generated as described [35].

### Animals

All animal studies were approved by the Government of Lower Franconia (Bezirksregierung Unterfranken; AZ 55.2–2531.01-84/14 and AZ 55.2–2531.01-62/12) and supervised by the Animal Welfare Committee and the Animal Welfare Officer of the University of Würzburg. The mice were handled by experienced animal care takers and veterinarians and were healthy until the experiments were performed. *5Htt*^*-/-*^ and *Unc13d*^*-/-*^mice were generated as previously described [36, 37]. Experiments were performed using 6 to 12 week old littermates from *5Htt*^*+/-*^ breeding pairs. Mice were anesthetized by intraperitoneal injection of a combination of midazolam/ medetomidine/ fentanyl or with isoflurane inhalation. The mice for *in vivo* experiments were anesthetized and reflexes were tested to ensure an appropriate level of anesthesia. After the end of the experiment the mice were directly killed under deep anesthesia.

### *In Vitro* Platelet Studies

Platelet preparation, aggregometry, flow cytometry of platelet count, activation and degranulation were performed as described previously [38] in the presence or absence of 10 μM extracellular 5-HT.

### Collagen and Thrombin Activated (COAT) Platelet Determination

Washed platelets (5 × 10^4^ platelets/μL) suspended in Tyrode’s-HEPES buffer containing 2 mM CaCl_2_ and were activated with the indicated agonists and concentrations. COAT platelets were determined by a co-staining approach using combinations of PE conjugated JON/A or Cy5 labeled fibrinogen with Annexin-V-DyLight 488.

### Fibrinogen Binding

Washed platelets (5 × 10^4^ platelets/μL) suspended in Tyrode’s-HEPES buffer containing 2 mM CaCl_2_ were activated with the indicated agonists and concentrations. Fibrinogen binding was determined using rabbit anti-fibrinogen-IgG-Cy5 for 15 min at 37°C. The samples were analyzed with a FACSCalibur (BD Biosciences) flow cytometer after stopping the reaction with Tyrode’s-HEPES containing 2 mM CaCl_2_.

### Microparticle Formation

Washed platelets (5 × 10^4^ platelets/μL) suspended in Tyrode’s-HEPES buffer containing 2 mM CaCl_2_ and were activated with the indicated agonists and stained with a combination of Annexin-V-DyLight 488 and PE conjugated JON6 antibody for 15 min at RT. The activated platelet population was determined by forward scatter and side scatter parameters and platelet derived microparticles were defined by their binding of Annexin-V-DyLight 488 and PE conjugated JON6 antibody.

### Intracellular Ca^2+^ Measurement

Platelets were washed and resuspended in modified Tyrode-HEPES buffer without CaCl_2_. Platelets were loaded with Fura-2/AM (5 μM) in the presence of Pluronic F-127 (0.2 μg/mL) for 30 min at 37°C. Labelled platelets were washed and resuspended in HBSS buffer containing 1 mM MgCl_2_ and with or without 1 mM CaCl_2_. Magnetically stirred platelets were activated with indicated agonists and fluorescence was determined with a PerkinElmer LS 55 fluorimeter with excitation at 340 and 380 nm and emission at 509 nm. Each measurement was calibrated using Triton X-100 and EGTA.

### Platelet Spreading on Fibrinogen

Coverslips were coated with fibrinogen (100 μg/mL, F4883, Sigma-Aldrich) and blocked with 1% BSA/PBS. After washing with Tyrodes-HEPES buffer, washed platelets (3 × 10^5^ platelets/μL) were either unstimulated or activated with 0.01 U/mL thrombin (10602400001, Roche). At the respective time point the reaction was stopped by addition 4% PFA/PBS and images taken with a Zeiss Axiovert 200 inverted microscope (100x/0.60 objective) equipped with a CoolSNAP-EZ camera (Visitron) and analyzed off-line using ImageJ software. Four different stages of platelet spreading were evaluated: stage 1—roundish; stage 2—filopodia only; stage 3—filopodia and lamellipodia; stage 4—fully spread.

### Measurements of Inositol Monophosphate (IP_1_)

Briefly, washed platelets (8×10^5^ platelets/μL) were prepared in phosphate-free Tyrode-HEPES buffer containing 50 mM LiCl and 1 mM Ca^2+^ or without extracellular CaCl_2_. Platelets were activated by platelet agonists for indicated time period and IP_1_ ELISA was performed according to the manufacturer’s protocol (Cisbio Bioassays).

### Measurement of 5-HT Content in Plasma and Platelets

Washed platelets (5 × 10^5^ platelets/μL) were activated for 5 min with the indicated agonists and concentrations. 5-HT levels in platelets were measured using a commercial 5-HT ELISA according to the manufacturer’s instructions (5-HT ELISA^Fast Track^, LDN GmbH & Co. KG). Plasma 5-HT levels were measured in PPP samples obtained from heparinized whole blood centrifuged twice at 2400 g for 10 min in the presence of 5 mM EDTA.

### Measurement of 5-HIAA in Urine

At midday, urine was collected from mice and used for the commercial ELISA (5-HIAA ELISA kit, LDN GmbH & Co. KG). 5-HIAA concentrations in the urine samples were analyzed according to the manufacturer’s instructions.

### Measurement of Melatonin in Blood Plasma

At midday, anesthetized mice were bled into 5 mM EDTA and plasma was obtained by centrifugation twice at 4°C for 10 min. Melatonin was extracted from the plasma and samples were diluted with 250 μL of 1x Stabilizer and used for the ELISA (Melatonin ELISA Kit, Enzo Life Sciences Inc.). The melatonin concentration in the extracted plasma samples was analyzed according to the manufacturer’s instructions.

### Tyrosine Phosphorylation Assay

Washed platelets (7 x10^5^ platelets/μL) containing 2 U/mL apyrase (Sigma-Aldrich), 10 μM indomethacin (Calbiochem) and 5 mM EDTA (AppliChem) were stimulated with 1 μg/mL CRP or rhodocytin (Rhd). Samples were collected at the indicated time points and lysed in an equal volume of 2% NP40 lysis buffer (300 mM NaCl, 20 mM TRIS, 2 mM EGTA, 2 mM EDTA, pH 7.5; 2% Igepal CA-630, 2 mM Na_3_VO_4_, 10 mM NaF). After denaturing the samples with reducing sample buffer, they were separated by SDS-PAGE and Western blots detected with phosphotyrosine specific 4G10 antibody (Millipore, 1:1000) and β-actin served as loading control (Sigma-Aldrich, 1:2500).

### Platelet Adhesion to Collagen under Flow

Heparinized whole blood was perfused over a collagen coated surface at 1000 s^-1^ to determine surface coverage and thrombus volume with or without co-infusion of 10 μM 5-HT, as well as PS exposure to determine procoagulant activity as previously described [38].

### Neutrophil Infiltration in Brain Sections

Cryo-embedded brains from mice with a 60 min reperfusion injury were cut into 10 μm-thick sections. Immunostaining of leukocytes on brain slides was performed according to the description of Schumann et al [39] with Ly6B.2 antibody (rat anti-mouse, MCA771G, AbD Serotec, 1:500). Five slices per animal were analyzed to count the total number of infiltrated leukocytes in ipsilesional hemisphere using 8 pictures per slide at a 20 fold magnification.

### Tail Bleeding Assay

One mm of the tail tip was cut and bleeding monitored by absorbing blood drops with a filter paper without contacting the wound site every 20 s until the time of cessation of blood flow (without exceeding 20 min total time of bleeding).

### Intravital Microscopy of Thrombus Formation in FeCl_3_-Injured Mesenteric Arterioles

Mesenteric arteries were exteriorized carefully and immobilized on a Petri dish. The injury of the arterioles was induced by application of a filter paper (3 mm^2^ triangular) saturated with 20% FeCl_3_. Thrombus formation of fluorescently labeled platelets (56F8 DyLight 488; anti-GPIX) was monitored up to 40 min or until occlusion (blood flow stopped for > 1 min). Images were recorded with a Zeiss Axiovert 200 inverted microscope (10x/0.60 objective) equipped with a CoolSNAP-EZ camera (Visitron).

### Mechanical Injury of the Abdominal Aorta

An ultrasonic flow probe (0.5 PSB 699; Transonic Systems) was placed around the abdominal aorta and thrombus formation was induced by firm compression of the aorta with forceps for app. 15 s. Blood flow was monitored for 30 min or until complete occlusion occurred (no blood flow for > 3 min).

### FeCl_3_ Injury of the Carotid Artery

An ultrasonic flow probe (0.5 PSB 699; Transonic Systems) was placed around the carotid artery and blood flow was measured. The injury of the vessel was induced by topical application of a saturated filter paper (0.5 x 1 mm) with 10% FeCl_3_ for 1.5 min. Blood flow was monitored until 30 min or complete occlusion occurred (no blood flow for > 3 min).

### Transient Middle Cerebral Artery Occlusion Model of Stroke

In the transient middle cerebral artery occlusion (tMCAO) model a filament was advanced through the right carotid artery up to the middle cerebral artery causing an ischemic stroke. After 60 min the filament was removed to allow reperfusion [40]. The extent of infarction was assessed 24 h after reperfusion on 2,3,5-triphenyltetrazolium chloride-stained brain sections. The mice were continuously monitored for 24 h after stroke and the neurological deficits and motor function were estimated using Bederson score [41] and the grip test [42]. The maximum tolerated symptom was Bederson score 4 (occasional unidirectional movement in circles: circling, additional rotation around the body’s longitudinal axis: spinning). At this level or after 24 h the mice were euthanized to avoid unnecessary suffering of the animals.

### Data Analysis

The presented results are mean ± SD from at least three independent experiments per group. Differences between control and *5Htt*^*-/-*^ mice were statistically analyzed using the Student’s t-test. For a test of independence, the two-tailed Fisher’s test for control vs the respective group was used for *in vivo* models. The Mann Whitney test, a non-parametric test was used for the Bederson score and the grip test. P-values < 0.05 were considered statistically significant (*P < 0.05; **P < 0.01; ***P < 0.001).

## Results

### Abolished 5-HT Uptake in 5Htt^-/-^ Platelets

**Fig 1:**
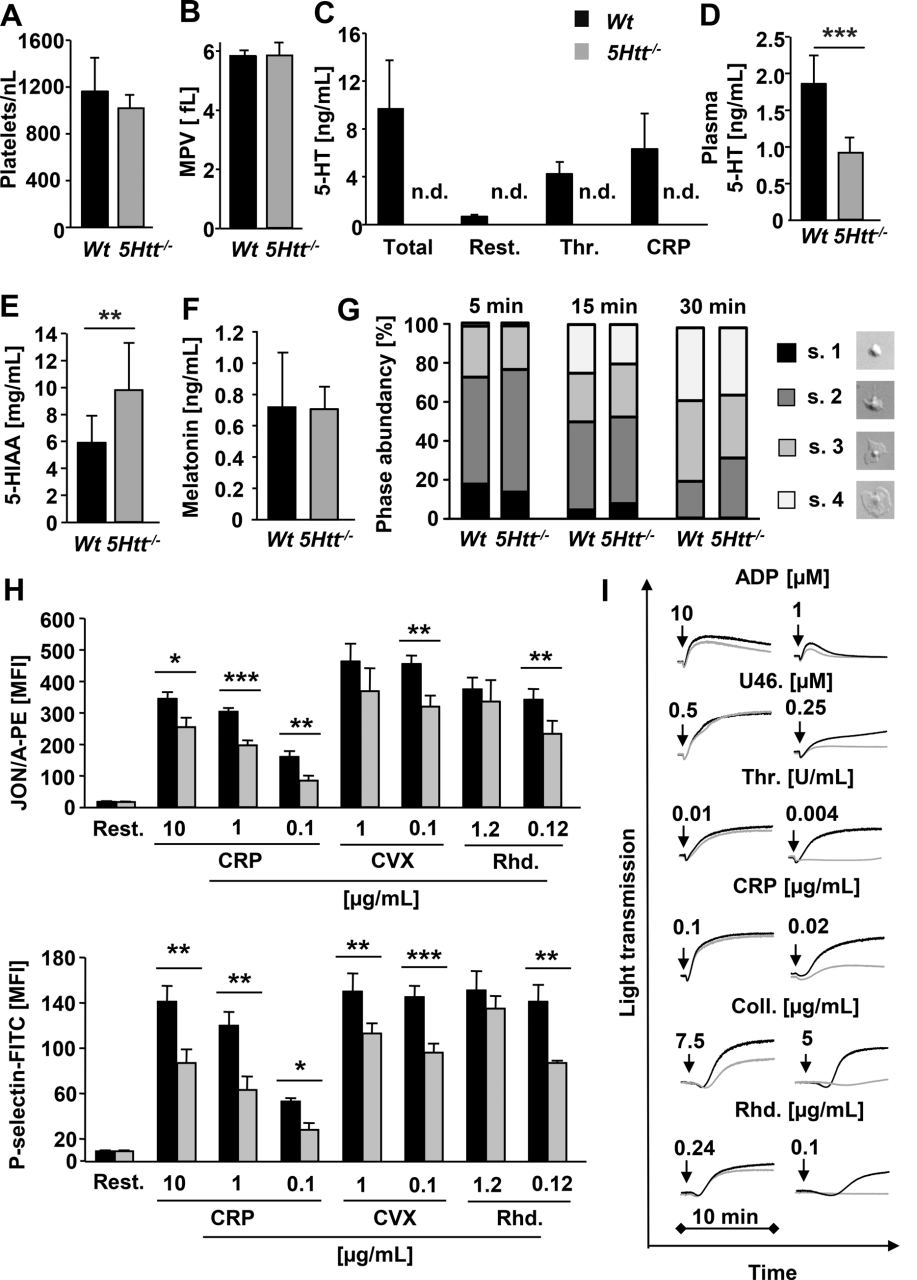
Abolished 5-HT uptake in *5Htt*^*-/-*^ platelets. Defective (hem)ITAM induced integrin activation, α-granule release and aggregation responses in *5Htt*^***-/-***^ platelets. (A) Platelet count and (B) size measured by FACS analysis or with a hematology analyzer (Sysmex). (C) Measurement of released platelet 5-HT before and after agonist dependent activation. 5-HT ELISA was performed with washed platelets of *Wt* and *5Htt*^*-/-*^ mice (n.d.: not detectable). Total 5-HT concentration was quantified in platelet lysates. (D) 5-HT concentration was measured in blood plasma with a 5-HT ELISA performed with platelet poor plasma (PPP) of *Wt* and *5Htt*^*-/-*^ mice. (E) 5-HIAA and (F) melatonin concentration was measured in urine and plasma samples of *Wt* and *5Htt*^*-/-*^, respectively with a 5-HIAA and melatonin ELISA. (G) Spreading of *Wt* and *5Htt*^*-/-*^ platelets on a fibrinogen coated surface in the presence of thrombin. Washed platelets of *Wt* and *5Htt*^*-/-*^ mice were allowed to spread for 5, 15 and 30 min after stimulation with 0.01 U/mL thrombin. Statistical evaluation of the percentage of spread platelets at different spreading stages. 1: roundish; 2: only filopodia; 3: filopodia and lamellipodia; 4: fully spread. (H) Flow cytometric analysis of *Wt* and *5Htt*^*-/-*^ platelets. Integrin αIIbβ3 activation (upper panel) and degranulation (lower panel) in response to the indicated agonists were measured on a FACSCalibur. Results presented as MFI ± SD (I) Aggregation responses of *5Htt*^*-/-*^ platelets (grey line) compared to *Wt* platelets (black line) measured by change in light transmission upon activation with indicated agonists. ADP measurements were performed in platelet rich plasma (PRP), whereas all others were performed in washed platelets (Thr: thrombin; U46.: stable thromboxane A_2_ analogue U46619; CRP: collagen-related peptide; Coll: HORM collagen; Rhd: rhodocytin; CVX: convulxin).

To study the function of 5HTT and platelet-stored 5-HT in thrombosis and hemostasis, we utilized mice genetically engineered to lack 5HTT (5*Htt*^*-/-*^) [37]. Platelet count (Fig 1A) and size (Fig 1B), expression of major surface glycoproteins (S1 Table) and basic blood cell parameters (S2 Table) were unaltered in *5Htt*^*-/-*^ mice indicating that 5HTT is not important for platelet production or general hematopoiesis. Measurement of the 5-HT concentration in blood plasma and platelets was performed by a 5-HT-ELISA. Release of 5-HT from knockout platelets was below the level of detection in all conditions tested (Fig 1C). These results showed that the 5-HT transporter 5HTT is essential for 5-HT uptake in platelets. This appears to be in sharp contrast to neurons, in which alternative compensatory 5-HT uptake mechanisms exist [43]. Additionally, we found that the 5-hydroxyindolacetic acid (5-HIAA) concentration, an important metabolite product of 5-HT, was increased in the urine of 5*Htt*^*-/-*^ mice (Fig 1E), whereas melatonin concentrations were normal in the 5*Htt*^*-/-*^ blood plasma (Fig 1F). In line with this, the concentration of 5-HT in the blood plasma was significantly reduced in *5Htt*^*-/-*^ mice (Fig 1D).

### Secreted Platelet 5-HT, but Not the 5HTT Transporter, Is Required for Maximal Platelet Responses to (Hem)ITAM Signaling

**Fig 2:**
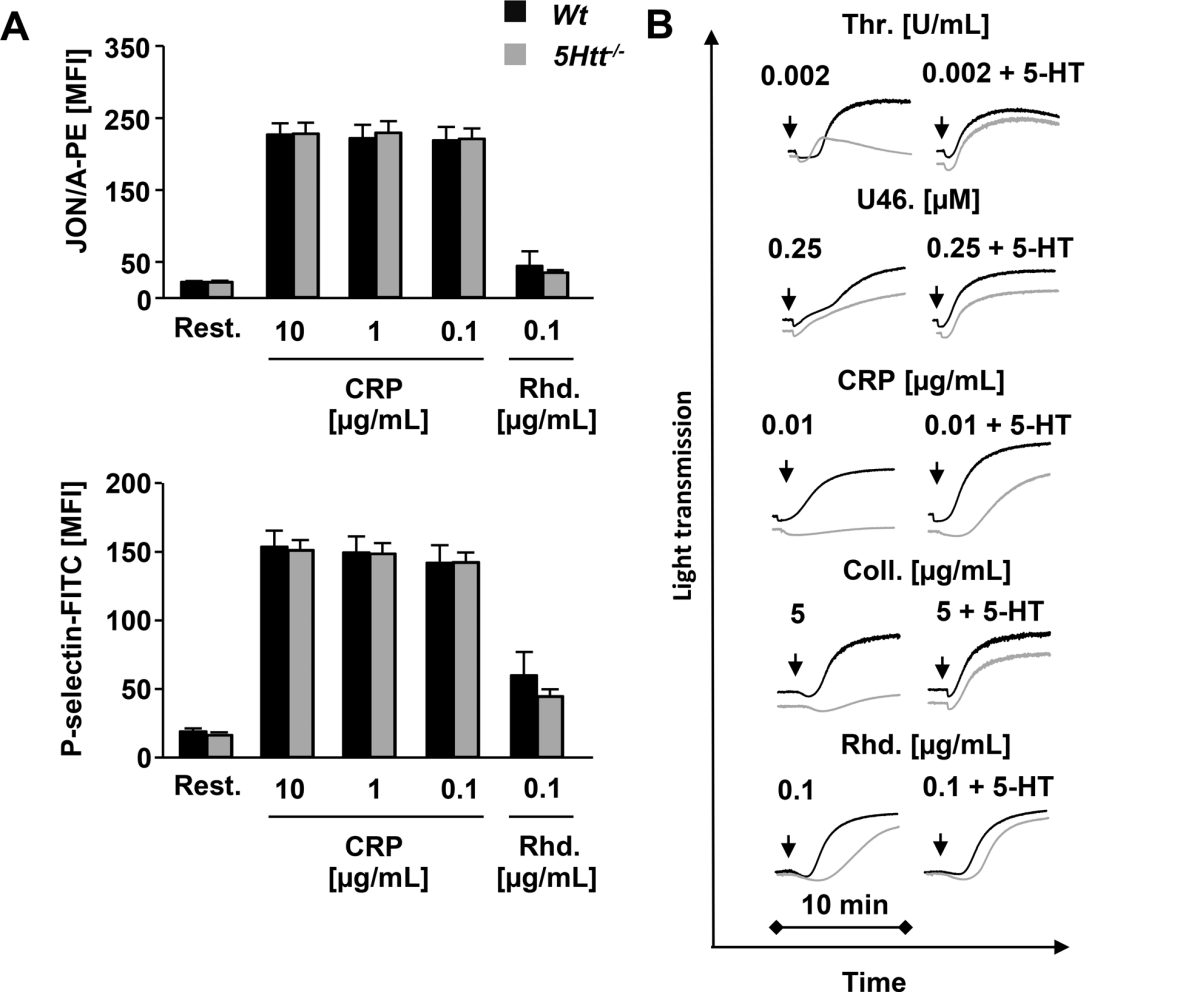
Defective (hem)ITAM signaling is rescued by addition of extracellular 5-HT. (A) Flow cytometric analysis of *Wt* and *5Htt*^*-/-*^ platelets in the presence of 10 μM 5-HT. Agonists and concentrations are indicated. Integrin αIIbβ3 activation was detected by JON/A-PE (upper panel) and degranulation was detected by anti-P-selectin-FITC as a marker of α-granule secretion (lower panel). Results are MFI ± SD. (B) Aggregation responses of *5Htt*^*-/-*^ platelets (grey line) compared to *Wt* platelets (black line) in the presence of 10 μM 5-HT upon activation with indicated agonists. Measurements were performed in washed platelets. Light transmission was recorded on a Fibrintimer 4-channel aggregometer and representative aggregation traces of at least 3 individual experiments are depicted. (Rest: resting platelets, Thr: thrombin, U46: U46619, a stable thromboxane A_2_ analogue, CRP: collagen-related peptide; CVX: convulxin, Rhd: rhodocytin, Coll.: HORM collagen).

5-HT is considered to be a “weak agonist” of platelets due to its inability to induce platelet aggregation by itself, but it is known to synergize with other signaling pathways and potentiate aggregation responses of other platelet agonists [44]. Furthermore, it has been shown that 5HTT itself directly interacts with integrin αIIbβ3 indicating a functional crosstalk between them [45]. To study the consequence of abolished 5HTT function and the loss of platelet stored 5-HT on outside-in signaling of αIIbβ3 integrins, platelet spreading assays were performed in the presence or absence of thrombin and no significant differences were observed under these conditions (Fig 1G and S1B Fig). In line with these results, fibrinogen binding was also found to be normal on the *5Htt*^*-/-*^ platelet surface after activation (S1C Fig). To study inside-out activation of αIIbβ3 integrins and degranulation, platelet responses to different agonists were monitored by flow cytometry. The contribution of platelet 5-HT or 5HTT to G-protein coupled receptor (GPCR) mediated platelet activation was not significant as *5Htt*^*-/-*^ platelet responses to higher concentrations of thrombin, and co-stimulation with ADP and U46619 were comparable to *Wt* platelets (S1D Fig). Of note, we observed a slight decrease in integrin activation and degranulation at threshold concentrations of thrombin and when ADP was used alone. In contrast to GPCR agonists, αIIbβ3 integrin activation and P-selectin surface exposure in response to agonists of the (hem)ITAM coupled receptors GPVI and CLEC-2 were significantly reduced in *5Htt*^*-/-*^ platelets revealing an important role for 5-HT and/or 5HTT in GPVI and CLEC-2 mediated platelet activation (Fig 1H). Similar defects in (hem)ITAM-mediated responses were also seen in aggregometry studies with *5Htt*^*-/-*^ platelets (Fig 1I). Importantly, surface expression of GPVI and CLEC-2 in *5Htt*^*-/-*^ platelets was not altered compared to *Wt* controls (S1 Table). Similarly, changes in protein tyrosine phosphorylation (S1E Fig) and PLC activity (S1F Fig) after GPVI or CLEC-2 stimulation were normal in *5Htt*^*-/-*^ platelets, as assessed by Western blotting and inositol monophosphate (IP_1_) ELISA, respectively. Therefore, we concluded that the defects in response to (hem)ITAM stimulation were downstream of the initial (hem)ITAM signaling cascade. To distinguish between the role of platelet released 5-HT and the 5HTT transporter itself, *5Htt*^*-/-*^ platelet activation and aggregation was repeated in the presence of 10 μM extracellular 5-HT. The addition of exogenous 5-HT normalized the defective integrin activation, P-selectin surface exposure (Fig 2A) and aggregation responses (Fig 2B) of *5Htt*^*-/-*^ platelets to (hem)ITAM agonists compared with *Wt* platelets. This restoration suggested that the transporter 5HTT itself does not play a role in platelet activation directly but rather the secretion of platelet stored 5-HT and subsequent initiation of 5HTR2A receptor signaling is required to amplify platelet (hem)ITAM mediated responses.

**Fig 3:**
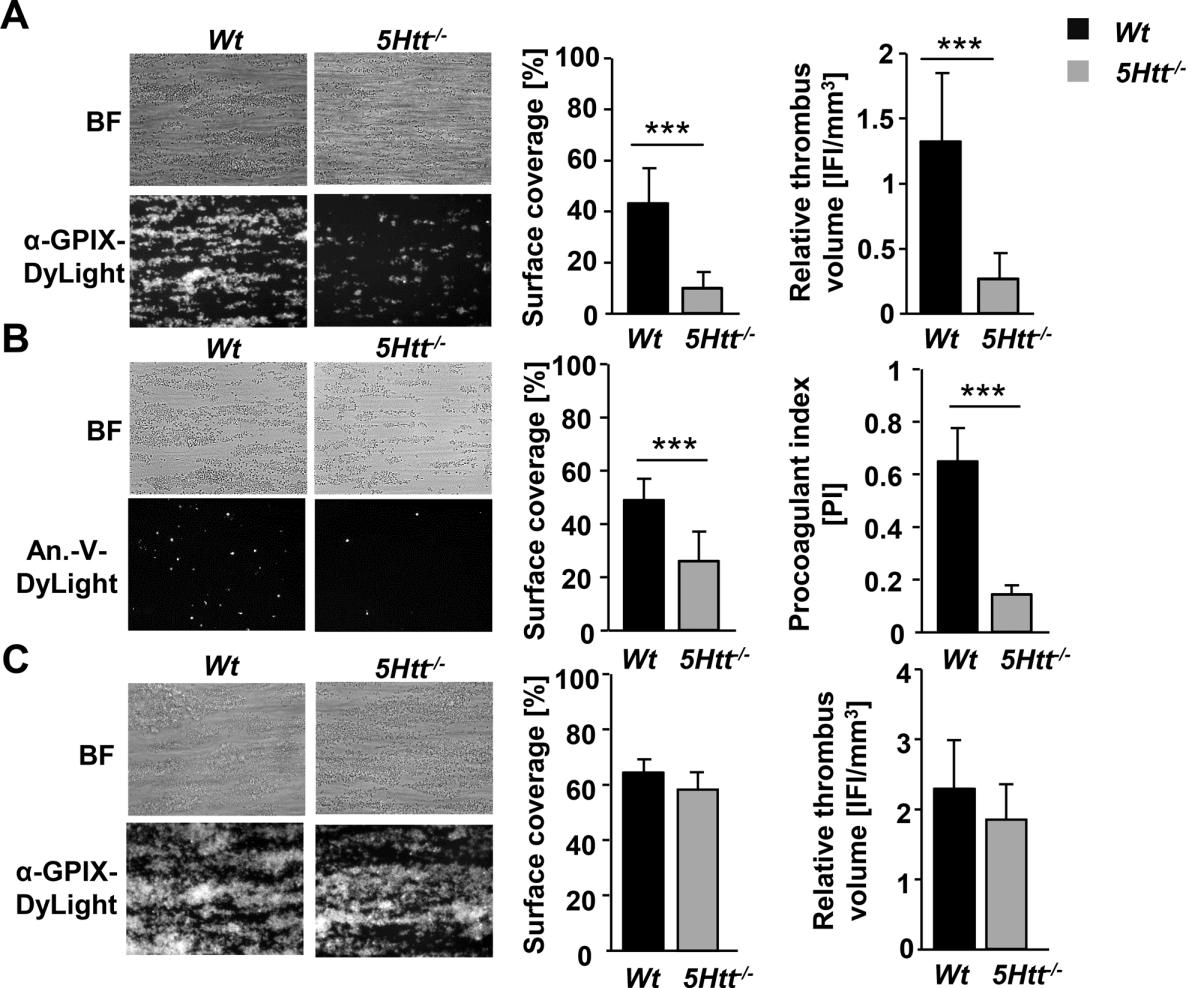
Altered collagen-induced adhesion and aggregate formation of *5Htt*^*-/-*^ platelets under flow. (A) Whole blood from *Wt* or *5Htt*^*-/-*^ mice was perfused over a collagen coated surface (0.2 mg/mL) at a shear rate of 1000 s^-1^. Representative brightfield (BF) and fluorescence (DyLight 488 conjugated anti-GPIX) images of aggregate formation on collagen after 4 minutes of perfusion time are shown. Mean surface coverage (%) ± SD and relative thrombus volume expressed as integrated fluorescence intensity (IFI) ± SD in both *Wt* and *5Htt*^*-/-*^ mice. (B) Impaired procoagulant activity of blood samples from *5Htt*^*-/-*^ mice. PS exposure was detected by Annexin-V-Dylight 488 under similar flow conditions as described above. (C) Restored adhesion and aggregate formation of *5Htt*^*-/-*^ platelets by co-infused 5-HT on a collagen coated surface under flow. Heparinized whole blood of either *Wt* or *5Htt*^*-/-*^ mice was perfused with 10 μM 5-HT over a collagen coated surface (0.2 mg/mL) at a shear rate of 1000 s^-1^.

To further investigate the importance of secreted platelet 5-HT in aggregation under shear flow conditions, heparinized blood of *Wt* or *5Htt*^*-/-*^ mice was perfused over a collagen coated surface at a shear rate of 1000 s^-1^ in the presence of a DyLight 488 conjugated anti-GPIX Ig derivative that labels platelets. *Wt* platelets initially adhered to the collagen surface and then recruited additional platelets from the flowing blood resulting in the formation of stable, three-dimensional thrombi that finally covered about 40% of the total surface area. In sharp contrast, in *5Htt*^*-/-*^ blood samples this process of aggregate formation was strongly reduced by almost three-fold (~15% surface coverage) (Fig 3A). In addition, the number of platelets exposing phosphatidylserine (PS) was dramatically reduced in the aggregates of the mutant animals resulting in a significantly reduced procoagulant index (Fig 3B). As for the other defects observed in *5Htt*^*-/-*^ platelets, a co-infusion of 10 μM 5-HT restored the aggregate formation and PS exposure in *5Htt*^*-/-*^ blood samples to the level of *Wt* samples (Fig 3C).

### 5-HT Potentiation of (Hem)ITAM Signaling Is Mediated by Store Operated Ca^2+^ Entry (SOCE)

**Fig 4:**
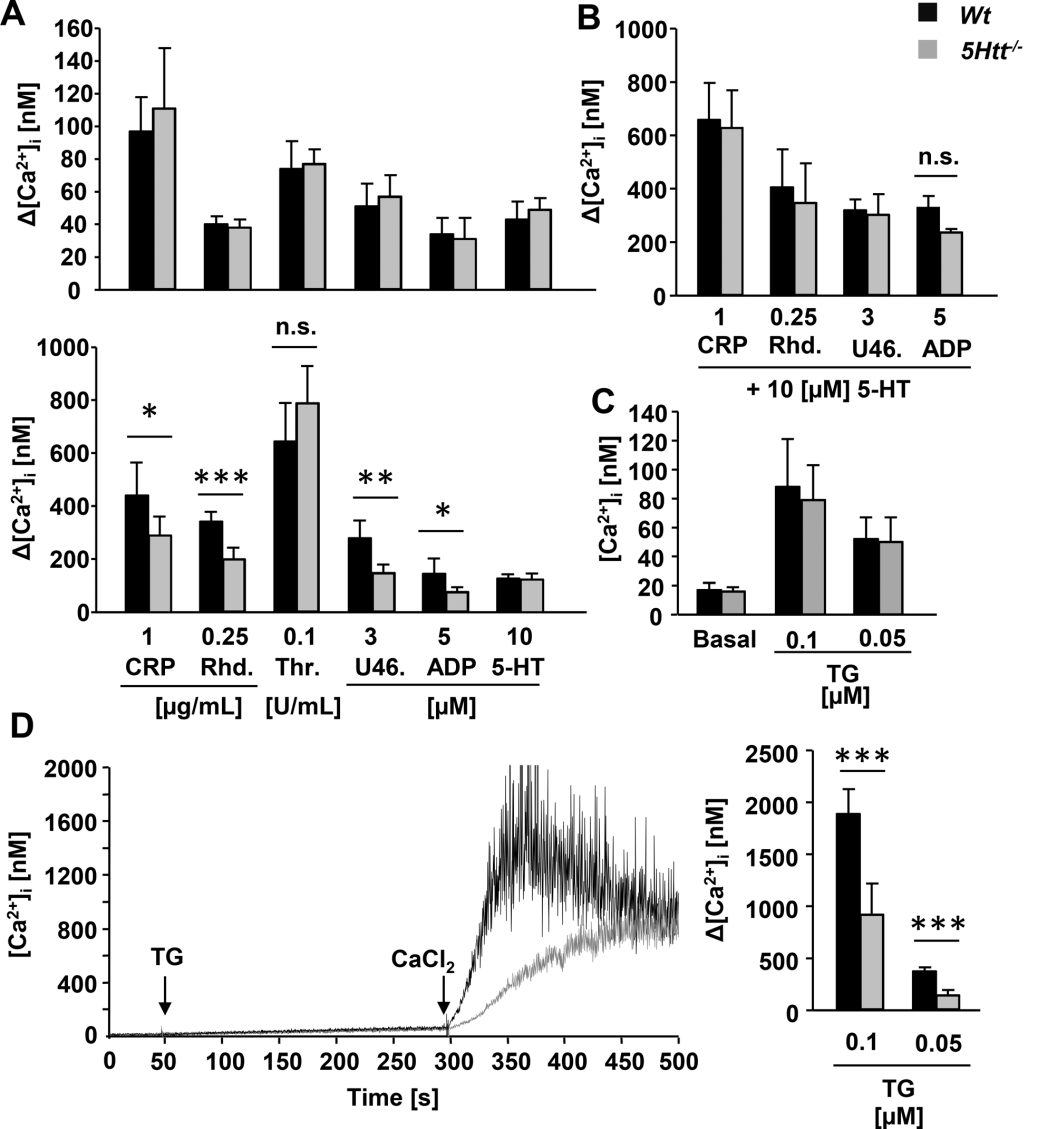
Store operated Ca^2+^ entry is reduced in *5Htt*^*-/-*^ platelets. (A) Normal Ca^2+^ store release (upper panel), but reduced Ca^2+^ response to platelet agonists in the presence of extracellular CaCl_2_ (lower panel) in *5Htt*^*-/-*^ platelets. Fura-2-loaded *Wt* (black bars) or *5Htt*^*-/-*^ (grey bars) platelets were stimulated with the indicated agonists in calcium-free medium or in the presence of extracellular 1 mM CaCl_2_, and [Ca^2+^]_i_ was monitored by fluorimetry. Representative measurements and maximal Δ[Ca^2+^]_i_ ± standard deviation (SD) are shown. (B) Platelets were labeled and stimulated under similar conditions as Fig 4A in the presence of 10 μM 5-HT. Representative measurements and maximal Δ[Ca^2+^]_i_ ± standard deviation (SD) are shown. (C) Unaltered cytoplasmic Ca^2+^ level in resting platelets and TG induced Ca^2+^ store release in *5Htt*^*-/-*^ platelets. (D) Reduced TG induced SOCE in *5Htt*^*-/-*^ platelets. SOCE was measured in fura-2-loaded platelets stimulated with 0.1 or 0.05 μM TG for 5 min followed by the addition of 1 mM extracellular CaCl_2_. Representative Ca^2+^ curves were indicated (black line: *Wt*; gray line: *5Htt*^*-/-*^). Maximal Δ[Ca^2+^]_i_ of SOCE were quantified. Data are mean ± SD.

A key factor in platelet integrin activation and degranulation is a sustained increase in cytoplasmic Ca^2+^ levels. 5-HT binds 5HTR2A on the platelet surface which modulates the Gq-PLCβ pathway and causes Ca^2+^ mobilization and protein kinase C (PKC) activation. Given that the initial signaling cascade downstream of (hem)ITAM coupled receptors (S1B Fig), including PLC activity (S1C Fig), and IP_3_ dependent Ca^2+^ store release (Fig 4A, upper panel) was unaffected in *5Htt*^*-/-*^ platelets, Ca^2+^ increase was measured in the presence of 1 mM CaCl_2_ (Fig 4B). In line with the functional defects in *5Htt*^*-/-*^ platelets, GPVI and CLEC-2 induced Ca^2+^ responses were significantly reduced in these cells (Fig 4A, lower panel). Surprisingly, ADP and U46619 mediated Ca^2+^ responses were also affected, whereas thrombin and 5-HT mediated Ca^2+^ increase were similar to *Wt* platelets (Fig 4A, lower panel). Importantly, in the presence of 10 μM extracellular 5-HT all Ca^2+^ responses were comparable between *5Htt*^*-/-*^ and *Wt* platelets (Fig 4B). This explains the reverted integrin activation and degranulation responses in 5-HT treated *5Htt*^*-/-*^ platelets and highlights the important autocrine role of secreted platelet 5-HT in the amplification of the Ca^2+^ entry mechanism. To further investigate the role of platelet 5-HT in the SOCE mechanism itself, thapsigargin (TG) as a sarcoplasmic/endoplasmic reticulum calcium ATPase (SERCA) pump inhibitor was used to deplete the Ca^2+^ store and activate SOCE [46]. Interestingly, basal Ca^2+^ level in the cytoplasm and TG induced Ca^2+^ store release were normal in the absence of extracellular CaCl_2_ (Fig 4C), but a significant reduction of SOCE was measured after addition of 1 mM CaCl_2_ in *5Htt*^*-/-*^ platelets compared to *Wt* platelets (Fig 4D). Taken together these results suggest that secreted platelet 5-HT accelerates Ca^2+^ store depletion via 5HTR2A-Gq-PLCβ signaling and amplifies Orai1 activity during the second phase of platelet activation.

### Impaired Thrombus Formation and Hemostasis in 5Htt^-/-^ Mice

**Fig 5:**
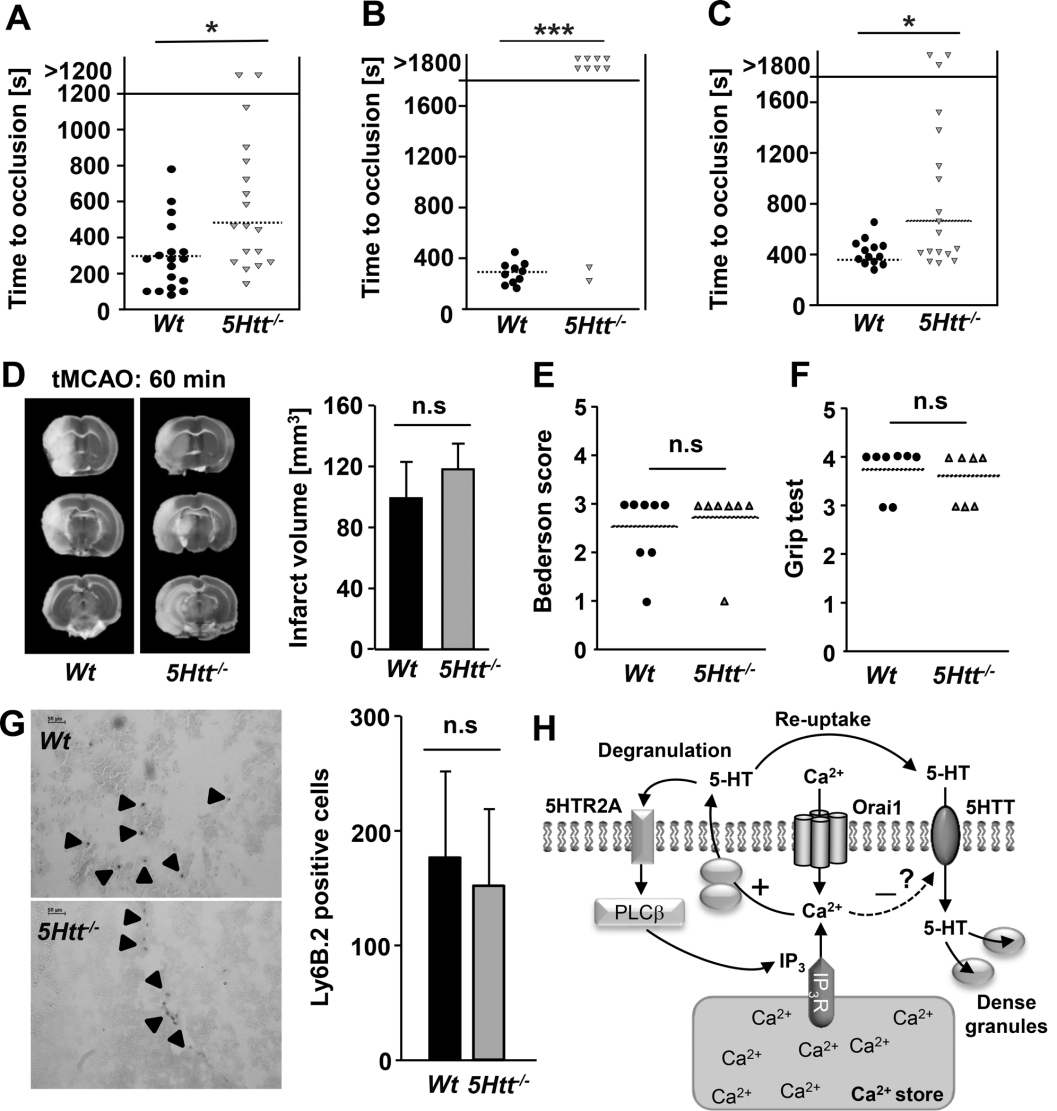
Altered hemostasis and thrombus formation in *5Htt*^*-/-*^ mice. (A) Prolonged tail bleeding times of *5Htt*^*-/-*^ mice. Each symbol represents one animal. (B-C) Impaired occlusion time of *5Htt*^*-/-*^ mice in models of arterial thrombosis. The abdominal aorta was injured by firm compression with forceps and blood flow was monitored by an ultrasonic flow probe until complete vessel occlusion occurred or for 30 min (B). Time to stable vessel occlusion of *Wt* and *5Htt*^*-/-*^ mice was determined. Each symbol represents one animal.(C) The right carotid artery was injured by the application of a FeCl_3_ soaked filter paper and blood flow was monitored for 30 min using a Doppler flow probe. Each symbol represents one animal. (D) Ischemic stroke development in *5Htt*^*-/-*^ mice using the transient middle cerebral artery occlusion (tMCAO) model with 60 min. Representative images of coronal brain sections (left) stained with 2,3,5-triphenyltetrazolium chloride after 24 hours are shown. Infarct volume was measured by planimetry 24 h after (right panel). (E) Bederson score and (F) grip test were determined 24 h after tMCAO. Each symbol represents one animal. Results represent mean ± SD. (G) Number of infiltrated leukocytes in the ischemic brain of *Wt* and *5Htt*^*-/-*^ mice. Representative pictures of Ly6B.2 immunostaining show similar number of leukocytes in both *Wt* and *5Htt*^*-/-*^ ischemic brains. (H) Proposed regulatory role of SOCE in serotonergic system in platelets. During platelet agonist dependent Ca^2+^ store depletion, STIM1 binds Ora1 and modulates SOCE. Enhanced Orai1 activity supports degranulation and 5-HT secretion. Secreted platelet 5-HT further amplifies Ca^2+^ store depletion through 5HTR2A-Gq-PLCβ signaling. At the same time, 5-HT uptake is inhibited by SOCE to keep secreted platelet 5-HT in the extracellular milieu. The functional link between Orai1 mediated SOCE and 5HTT is so far unknown. Abbreviations: phospholipase C (PLC), inositol trisphosphate receptor (IP_3_R), inositol trisphosphate (IP_3_), 5-HT transporter (5HTT), serotonin (5-HT), serotonin receptor (5HTR2A).

The *in vitro* functional analysis of *5Htt*^*-/-*^ platelets revealed a role for platelet stored 5-HT release and its subsequent signaling in potentiation of Ca^2+^ entry initiated by (hem)ITAM signaling. To address the importance of this 5-HT mediated feed-forward pathway in the more complex processes of thrombosis and hemostasis, *5Htt*^*-/-*^ mice were subjected to bleeding time analysis and a model of *in vivo* thrombus formation. Prolonged bleeding times were observed in *5Htt*^*-/-*^ mice (Fig 5A) (*Wt*: 291 ± 194 s vs. *5Htt*^*-/-*^: 482 ± 279 s) reflecting the increased bleeding risk described to occur upon SSRI treatment. Strikingly, in comparison to this relatively mild hemostatic defect, 80% of *5Htt*^*-/-*^ mice were not able to form occlusive thrombi in response to mechanical injury of the abdominal aorta within the observed time period (Fig 5B), whereas 100% of the *Wt* control did. Platelet activation with both GPVI and protease-activated receptor (PAR) agonists results in the production of highly PS positive platelets, the so-called collagen and thrombin activated (COAT) platelets [47, 48]. 5-HT has been proposed to be involved in COAT platelet formation thereby enhancing microparticle formation, coagulation and thrombus stabilization [49–51]. On the surface of COAT platelets, coagulation factors and fibrinogen are covalently linked to 5-HT (serotonylation) which stabilizes these proteins for the coagulation cascade or integrin activation, respectively. To test whether COAT platelet and microparticle formation are impaired in *5Htt*^*-/-*^ mice, platelets were stimulated with both convulxin and thrombin. Remarkably, no differences in the numbers of COAT platelets were observed between *Wt* and mutant samples (S2A Fig). Microparticle formation was also found to be normal in *5Htt*^*-/-*^ mice (S2B Fig) thus excluding a role of 5-HT in these processes.

### Platelet-Released 5-HT Is Not a Critical Factor in Experimental Ischemic Stroke

Platelets play a unique role in the initiation of brain infarct growth after transient ischemia in a process termed thrombo-inflammation. This process, as it is currently understood, involves a complex interplay between platelets and immune cells but is not dependent on platelet aggregation. Interestingly, SSRI treatment of stroke patients is described to enhance brain function recovery, indicating a therapeutic benefit of the direct blockade of 5HTT function [52–54]. Given the distinct mechanisms thought to be involved in thrombo-inflammation and the described benefit of SSRI treatment of stroke patients, *5Htt*^***-/-***^ mice were subjected to the tMCAO model of ischemic stroke, where blood flow to the middle cerebral artery is blocked for 60 min before reperfusion, to further investigate the direct role of 5HTT and platelet stored 5-HT under ischemic conditions. Although *5Htt*^***-/-***^ mice were moderately protected in a carotid artery model of thrombosis (Fig 5C), unexpectedly, these mice developed large brain infarcts following tMCAO (Fig 5D) and had a neurological outcome indistinguishable to that of *Wt* mice as assessed by the Bederson score (Fig 5E) and grip test (Fig 5F). Of note, leukocyte infiltration into the infarct area was not elevated in *5Htt*^***-/-***^ brain tissue (Fig 5G), and no significant difference between *Wt* and *5Htt*^***-/-***^ mice were detected in the tMCAO model after 30 min of blood flow blockage (S2C–S2E Fig).

## Discussion

In human platelets, several 5-HT transporters have been detected at the mRNA level including 5Htt (*Slc6a4*) and DAT (*Slc6a3*) [55]. In contrast to a previous observation, genetic ablation of *5Htt* in mice completely blocks 5-HT uptake in platelets (Fig 1C). Therefore we conclude that other pathways cannot compensate the lack of 5HTT function in these cells.

In β3 integrin knockout platelets 5-HT uptake was strongly reduced indicating a functional crosstalk between 5HTT and β3 integrin [45]. Our finding that the αIIbβ3 activation defect in response to GPVI or CLEC-2 stimulation in *5Htt*^*-/-*^ platelets (Fig 1H) was fully rescued in the presence of extracellular 5-HT (Fig 2A) clearly demonstrates that the physical interaction between 5HTT and β3 is not essential for integrin activation. To further support this, fibrinogen binding to integrins and outside-in signaling of αIIbβ3 integrin on fibrinogen were normal during spreading of *5Htt*^*-/-*^ platelets (Fig 1G and S1B Fig). Therefore, we assume that the observed activation defect is due the lack of the secreted platelet 5-HT which triggers “inside-out” activation of integrins through Ca^2+^ dependent (CalDAGGEF) and independent pathways (PKC) which induced by 5HTR2A-Gq-PLCβ signaling.

Although 5-HT significantly amplifies platelet reactivity through 5HTR2A signaling and induces platelet shape change, it has been proposed to play a minor role in aggregate formation, since 5-HT alone cannot induce aggregation responses. However, we found that aggregation responses to collagen or rhodocytin were strongly reduced in *5Htt*^*-/-*^ platelets (Fig 1I) indicating an important role of 5-HT in these signaling pathways. Indeed, the blockade of 5HTT with the SSRI citalopram reduces the aggregation response to collagen in human platelets [18] due to reduced Syk phosphorylation in the GPVI signalosome. Additionally, Syk can bind and phosphorylate 5HTT. These results supported the idea that 5HTT and Syk interaction might regulate the GPVI complex. To test this concept, we activated *5Htt*^*-/-*^ platelets in different experimental conditions to dissect the possible role of 5HTT in (hem)ITAM signaling. Surprisingly, we could not find any abnormalities in the initial phase of tyrosine phosphorylation cascade of the GPVI or CLEC-2 signalosomes (S1E Fig:). To demonstrate the indirect role of 5HTT in platelet signaling, we could completely rescue GPVI or CLEC-2 mediated Ca^2+^ influx (Fig 4B), integrin activation, degranulation and aggregation defects (Fig 2A and 2B) in *5Htt*^*-/-*^ platelets using extracellular 5-HT. Additionally, strongly reduced thrombus area and volume were observed at high shear flow conditions on a collagen coated surface in *5Htt*^*-/-*^ blood which was completely rescued by 5-HT co-infusion (Fig 3C). Taken these results together, we conclude that although Syk and 5HTT interaction is dispensable in the initial phase of GPVI or CLEC-2 activation, modulation of Syk activity by (hem)ITAM signaling seems to play an important regulatory role in 5-HT uptake mechanism as previously suggested to occur in human platelets [17].

During platelet activation both integrin activation and degranulation requires threshold levels of [Ca^2+^]_I_ mediated by SOCE [12]. Orai1 induced SOCE is triggered through the release of Ca^2+^ from intracellular stores which are tightly regulated by functional coupling of activated stromal interaction molecule 1 (STIM1) to the Orai1 complex [46]. Interestingly, we found a strongly reduced SOCE in *5Htt*^*-/-*^ platelets. In agreement with published results [12, 46], 5-HT can further enhance SOCE through binding to 5HTR2A which activates Gq-PLCβ mediated Ca^2+^ store release, generating a second activation step of STIM1 which is required for full activation of Orai1 mediated SOCE during degranulation. To distinguish the role of intracellular and extracellular 5-HT in SOCE activation, *Unc13d*^*-/-*^ platelets were used in which dense granule release (ATP and 5-HT secretion) is abrogated [36]. Similarly to *5Htt*^*-/-*^ platelets, TG induced SOCE was reduced in *Unc13d*^*-/-*^ platelets indicating a dispensable role of intracellular 5-HT in SOCE activation (S2F Fig) and underscoring the role of secreted platelet 5-HT in the second phase of Orai1 activation. Additionally, it has been shown that TG induced SOCE strongly inhibits 5-HT uptake in human platelets [56, 57]. This could be an important step to keep 5-HT outside of platelets and permanently activate 5HTR2A on the platelet surface. Therefore 5-HT cannot circulate between the extracellular space and the platelet cytosol after SOCE activation (Fig 5H).

At sites of vascular injury, 5-HT release by activated platelets is clinically relevant to induce acute thrombotic events [58, 59] by promoting vasoconstriction and activation of platelets. The long term use of SSRI has been shown to decrease the 5-HT concentration in human platelets and thereby exert a significant anti-thrombotic effect [60, 61]. In line with this, 5-HT uptake and release was completely abolished in *5Htt*^*-/-*^ mice (Fig 1C). Since platelets are the major store of 5-HT in the blood, blocking the 5-HT uptake mechanism in the periphery should increase 5-HT level in the blood, as earlier observed in *5Htt*^*-/-*^ brain [62]. Surprisingly, however, we found strongly reduced 5-HT levels in the blood plasma of *5Htt*^*-/-*^ mice (Fig 1D). We speculated that functional blockage of 5HTT in the periphery might induce abnormal 5-HT metabolism in the vascular system thereby increasing the amount of metabolic products of 5-HT in the blood or urine. There are two major routes of 5-HT metabolism in the body which convert 5-HT to melatonin and 5-HIAA. We tested both routes and found only elevated urinary 5-HIAA levels in *5Htt*^*-/-*^ mice (Fig 1E) supporting an important role for platelet 5-HT uptake in the control of systemic 5-HT metabolic cycles.

In the carotid artery of rat, a 15-fold increased in 5-HT levels were detected upon injury indicating a potential role of secreted platelet 5-HT during thrombus formation [63]. In agreement with this result, complete block of 5-HT synthesis in the periphery protects mice in the mesenteric artery model of thrombosis [64]. Furthermore, a direct effect of 5-HT on vascular smooth muscle was found to activate vessel wall contraction [59, 65]. Based on our results showing defective arterial thrombus formation in *5Htt*^*-/-*^ (Fig 5B and 5C) we assume that secreted platelet 5-HT may have a paracrine effect on neighboring platelets and other cells at the site of vessel wall injury. Furthermore, our results clearly show an additional autocrine effect through 5HTR2A activation which strongly potentiates SOCE activity. The lack of both paracrine and autocrine effects of 5-HT may explain the observed thrombus instability in *5Htt*^*-/-*^ mice.

Several studies suggest that long term inhibition of 5-HT uptake systems with SSRI increases the risk of bleeding complication in humans [66–68]. Prolonged bleeding times were observed in *Tph1*^*-/-*^ [64] and *5Htt*^*-/-*^ mice (Fig 5A) and this alteration was rescued by addition of extracellular 5-HT into the blood of *Tph1*^*-/-*^ mice [64]. In line with this, Ziu et al. showed that 5-HT infusion with mini-pumps generated hyperreactive platelets in *Wt* mice with reduced bleeding times and occlusion times of the carotid arteries [26]. Interestingly, 5-HT has been proposed to be involved in COAT platelet formation in which adhesive and procoagulant proteins are covalently linked to 5-HT by transglutaminase and regulates hemostasis [48]. SSRI treatment decreased the ability to generate COAT platelets in humans [47, 48]. In contrast to this hypothesis, however, COAT platelet and microparticle productions were normal in *5Htt*^*-/-*^ mice (S2A and S2B Fig) which precludes a fundamental role of secreted platelet 5-HT in these processes.

Stroke patients often suffer from post-stroke depression [69]. During SSRI treatment, enhanced functional recovery is observed in these patients [52–54]. Neuroblast proliferation and cell migration have been shown to be enhanced and associated with increased microvessel density during SSRI treatment, explaining the possible role of 5-HT uptake system in tissue repair after ischemic insults [52]. However, using a permanent occlusion model of branches of the middle cerebral artery, SSRI treatment did not reduce infarct size or cerebral edema in mice [52] suggesting that SSRI treatment cannot protect neurons or other cells in the brain during ischemic insults. Therefore, we conclude that SSRI treatment may have a long-term effect in the neurons of ischemic brain which positively influences post-stroke recovery. Using our tMCAO model with different time periods of the middle cerebral artery occlusion we could not observe major differences between *Wt* and *5Htt*^*-/-*^ mice (Fig 5D–5F and S2C–S2E Fig). The minor increase seen in infarct size did not alter the functional outcome measured 24 hours after reperfusion (Fig 5D–5F). It is important to note that in the brains of *5Htt*^*-/-*^ mice elevated extracellular 5-HT levels were detected. Given the disruption of the blood brain barrier in the acute and subacute phase after ischemic stroke, elevated level of 5-HT in the brain may induce infiltration of detrimental inflammatory cells from the blood into the brain parenchyma. However, we could not observe increased numbers of leukocytes in the ischemic infarct area of *5Htt*^*-/-*^ mice (Fig 5G).Further characterization of *5Htt*^*-/-*^ mice is necessary to understand the role of 5HT in the context of thrombo-inflammation during stroke and infarct progression.

In summary, our results identify 5HTT as the major route of 5-HT uptake in platelets and show that platelet stored 5-HT is critical for hemostasis and thrombosis, but not for cerebral infarct progression in a model of experimental stroke.

## Supporting Information

S1 Fig (A) Agonists dependent Ca^2+^ responses in *Wt* and *5Htt*^*-/-*^ platelets. Representative Ca^2+^ curves were shown (black line: *Wt*; gray line: *5Htt*^*-/-*^) (B) Spreading of *Wt* and *5Htt*^*-/-*^ platelets on a fibrinogen coated surface in the absence of thrombin. Statistical evaluation of the percentage of spread platelets at different spreading stages. 1: roundish; 2: only filopodia; 3: filopodia and lamellipodia; 4: fully spread. (C) Fibrinogen binding on the surface of *Wt* and *5Htt*^*-/-*^ platelets upon platelet activation. Washed platelets were stained with anti-fibrinogen-Cy5 in the presence of the indicated platelet agonists. Data are presented as mean MFI ± SD. Agonists and concentrations were indicated. MFI: mean fluorescence intensity (D) Flow cytometric analysis of *Wt* and *5Htt*^*-/-*^ platelets for integrin αIIbβ3 activation (left panel) and degranulation-dependent P-selectin exposure (right panel) in response to GPCR agonists. Results are mean fluorescence intensity (MFI) ± SD. (E) Western blot analysis of tyrosine phosphorylation of *Wt* and *5Htt*^*-/-*^ platelets shows no alteration in the phosphorylation status of several proteins after activation with CRP (1 μg/mL, left panel) or rhodocytin (2 μg/mL, right panel) in the presence of second wave mediator inhibitors (apyrase, indomethacin, EDTA). (F) Unaltered PLC activity in *5Htt*^*-/-*^ platelets. IP_1_-ELISA was performed in the presence of second wave mediator inhibitors and showed normal IP1 production in *5Htt*^*-/-*^ platelets.
(TIF)

S2 Fig (A) Quantification of COAT platelets in resting and activated conditions. Washed platelets were stained with JON/A-PE and Annexin-V-Dylight 488 in the presence of the indicated platelet agonists. A Ca^2+^ ionophore (A23187) was used as positive control. Percentage of all cells ± SD is indicated. (B) Analysis of microparticle formation from washed *Wt* and *5Htt*^*-/-*^ platelets stained with JON6-PE (anti-αIIbβ3) and Annexin-V-Dylight 488 after platelet activation using the indicated platelet agonists. (C) Ischemic stroke development in *5Htt*^*-/-*^ mice after the 30 min of transient middle cerebral artery occlusion (tMCAO) model. Representative images of coronal brain sections (left) stained with TTC after 24 hours are shown. Infarct volume was measured by planimetry 24 h after tMCAO (right panel). (D) Bederson score and (E) grip test were determined 24 h after tMCAO. Each symbol represents one animal. Results represent mean ± SD. (F) Reduced TG mediated SOCE in *Unc13*^-/-^ platelets. SOCE was measured in fura-2-loaded platelets stimulated with 0.1 μM TG for 5 min followed by the addition of 1 mM extracellular CaCl2. Maximal Δ[Ca2+]_i_ of SOCE was quantified. Data are presented as mean ± SD.
(TIF)

S1 Table Normal glycoprotein expression in *5Htt*^*-/-*^ platelets determined by flow cytometry. Diluted whole blood from *Wt* and *5Htt*^*-/-*^ mice was incubated with FITC-labeled antibodies as indicated for 15 min at RT and platelets were analyzed immediately. Data are expressed as mean fluorescence intensity ± SD.
(TIF)

S2 Table Normal hematology parameters of *5Htt*^*-/-*^ mice. Quantification of blood parameters performed with a hematology analyzer (Sysmex). White blood cell count (WBC), red blood cells (RBC), hemoglobin (HGB), hematocrit value (HCT), mean RBC volume (MCV), mean RBC hemoglobin (MCH), mean RBC hemoglobin concentration (MCHC).
(TIF)

## Abbreviations

ADP: adenosine diphosphate
BSA: bovine serum albumin
ORAI1: calcium release-activated calcium modulator 1
coll.: collagen
COAT: collagen and thrombin activated
CRP: collagen-related peptide
CLEC-2: C-type lectin-like receptor 2
EGTA: ethylene glycol tetraacetic acid
EDTA: ethylenediaminetetraacetic acid
FITC: fluorescein isothiocyanate
GPVI: glycoprotein VI
GPCR: G-protein coupled receptor
IP_1_: inositol monophosphate
IP_3_: inositol-1,4,5-triphosphate
PE: phycoerythrin
PS: phosphatidylserine
PKC: phosphokinase C
PLC: phospholipase C
PPP: platelet poor plasma
PRP: platelet-rich plasma
Rhd: rhodocytin
RT: room temperature
SERCA: sarcoplasmic/ endoplasmic reticulum calcium ATPase
SSRI: selective serotonin re-uptake inhibitors
5-HIAA: 5-hydroxyindolacetic acid
5-HT: serotonin
5HTR2A: serotonin receptor 2A
5HTT: serotonin transporter
*5Htt^-/-^*: serotonin transporter knockout mouse
SOCE: store operated Ca^2+^ entry
STIM1: stromal interaction molecule 1
TG: thapsigargin
TxA2: thromboxane A_2_
tMCAO: transient middle cerebral artery occlusion
TPH1: tryptophan hydroxylase 1
ITAM: immunoreceptor tyrosine-based activation motif
