# Supplementary figures and images for: Partially Defective Store Operated Calcium Entry and Hem(ITAM) Signaling in Platelets of Serotonin Transporter Deficient Mice
Source: PLoS One. 2016 Jan 22;11(1):e0147664. doi: 10.1371/journal.pone.0147664 (PMC4723080; doi:10.1371/journal.pone.0147664)

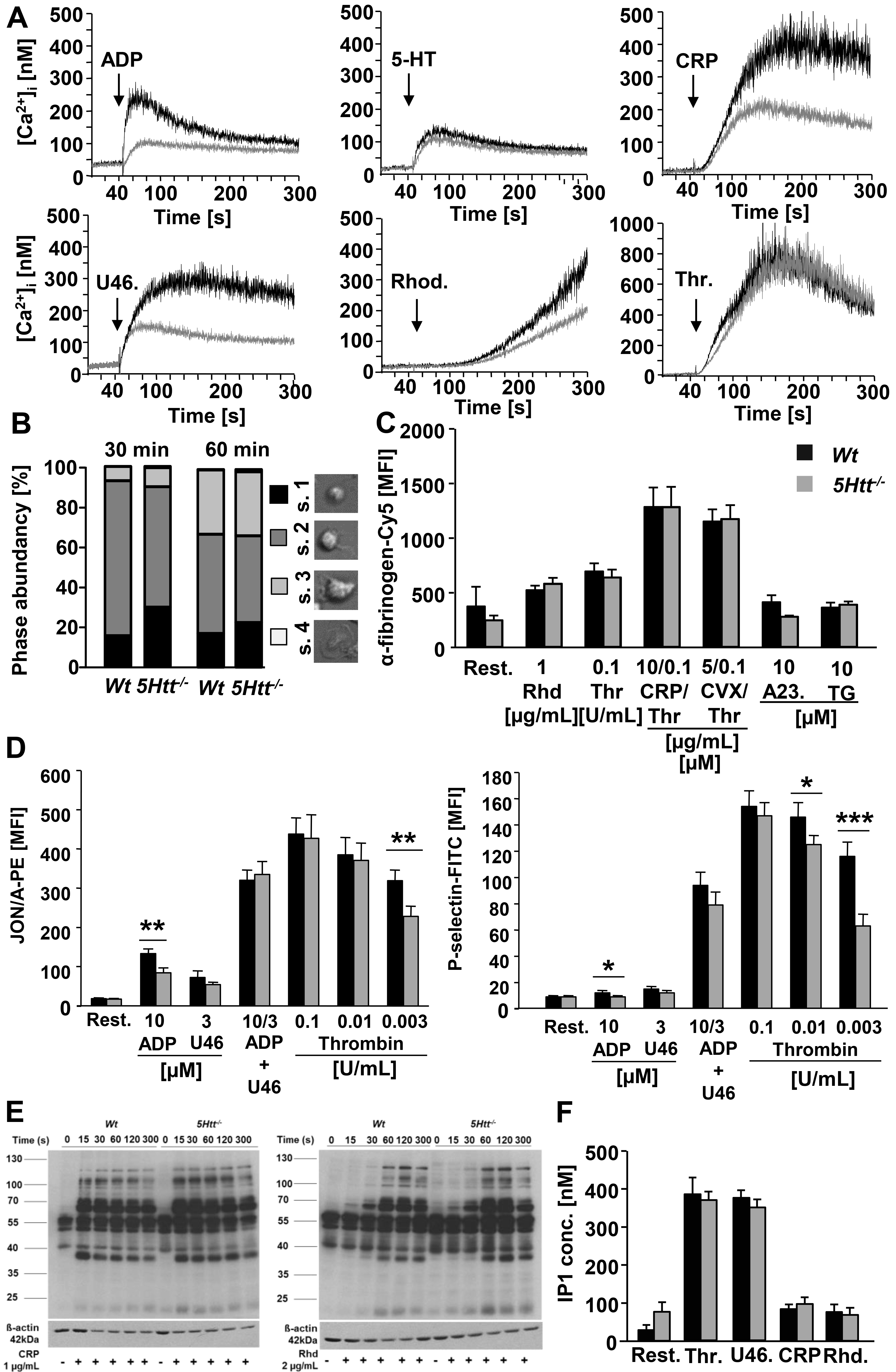

Supplement: S1 Fig — IP1-ELISA was performed in the presence of second wave mediator inhibitors and showed normal IP1 production in 5Htt-/- platelets. (TIF) [file pone.0147664.s001.tif]

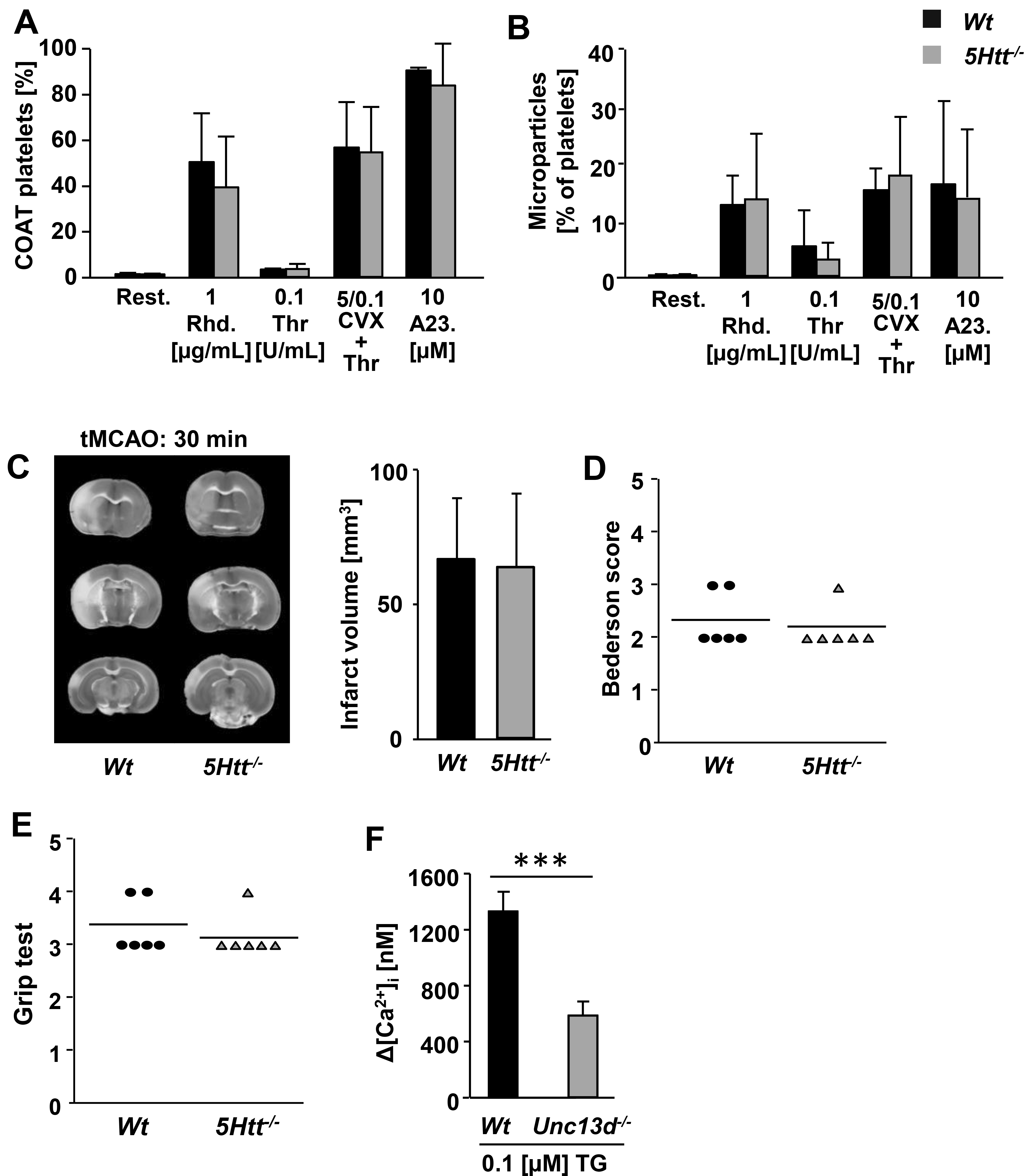

Supplement: S2 Fig — SOCE was measured in fura-2-loaded platelets stimulated with 0.1 μM TG for 5 min followed by the addition of 1 mM extracellular CaCl2. Maximal Δ[Ca2+]i of SOCE was quantified. Data are presented as mean ± SD. (TIF) [file pone.0147664.s002.tif]

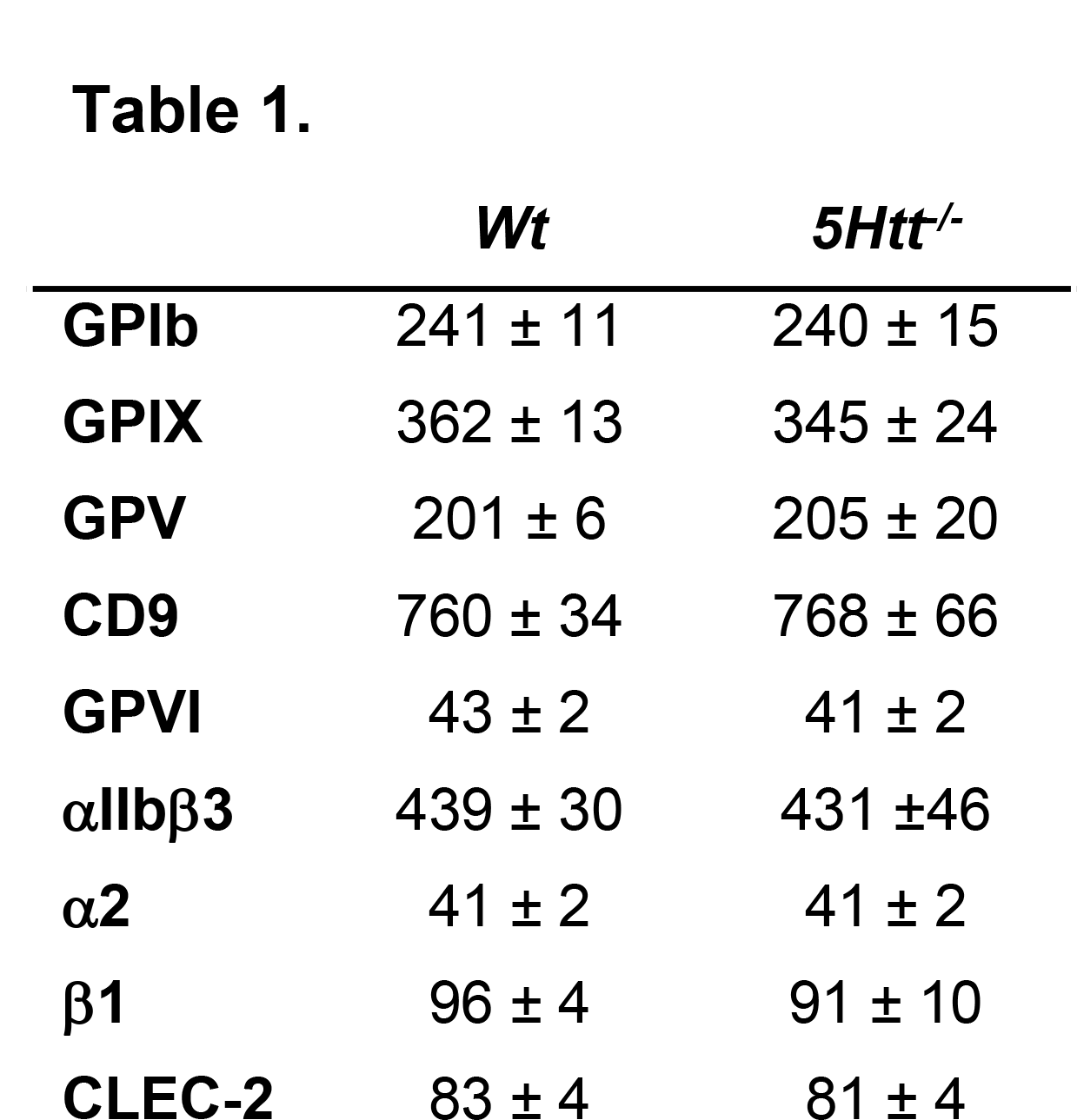

Supplement: S1 Table — Diluted whole blood from Wt and 5Htt-/- mice was incubated with FITC-labeled antibodies as indicated for 15 min at RT and platelets were analyzed immediately. Data are expressed as mean fluorescence intensity ± SD. (TIF) [file pone.0147664.s003.tif]

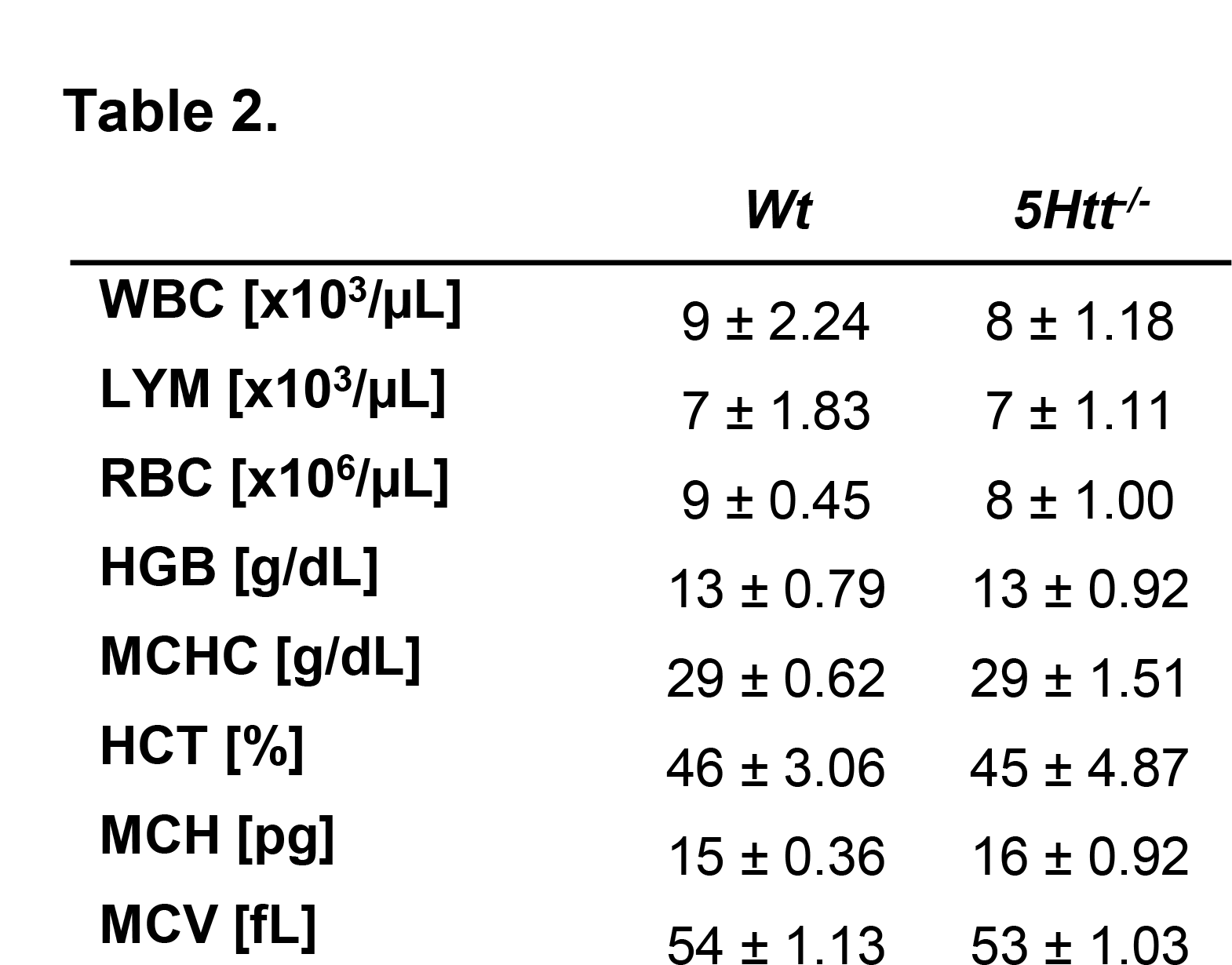

Supplement: S2 Table — Quantification of blood parameters performed with a hematology analyzer (Sysmex). White blood cell count (WBC), red blood cells (RBC), hemoglobin (HGB), hematocrit value (HCT), mean RBC volume (MCV), mean RBC hemoglobin (MCH), mean RBC hemoglobin concentration (MCHC). (TIF) [file pone.0147664.s004.tif]
